# Supplementary figures and images for: Pro-survival roles for p21(Cip1/Waf1) in non-small cell lung cancer
Source: Br J Cancer. 2024 Dec 20;132(5):421–37. doi: 10.1038/s41416-024-02928-9 (PMC11876327; doi:10.1038/s41416-024-02928-9)

TP53 WT

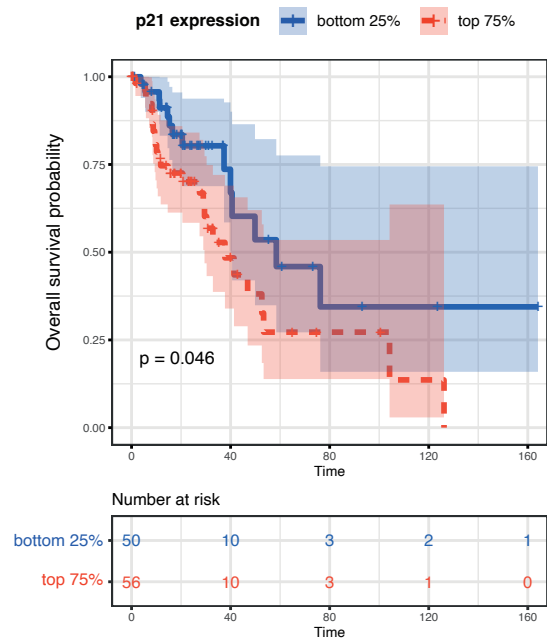

TP53 MUT

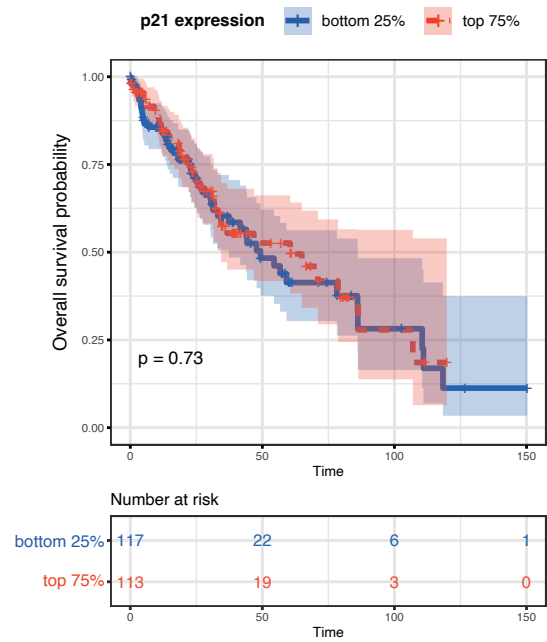

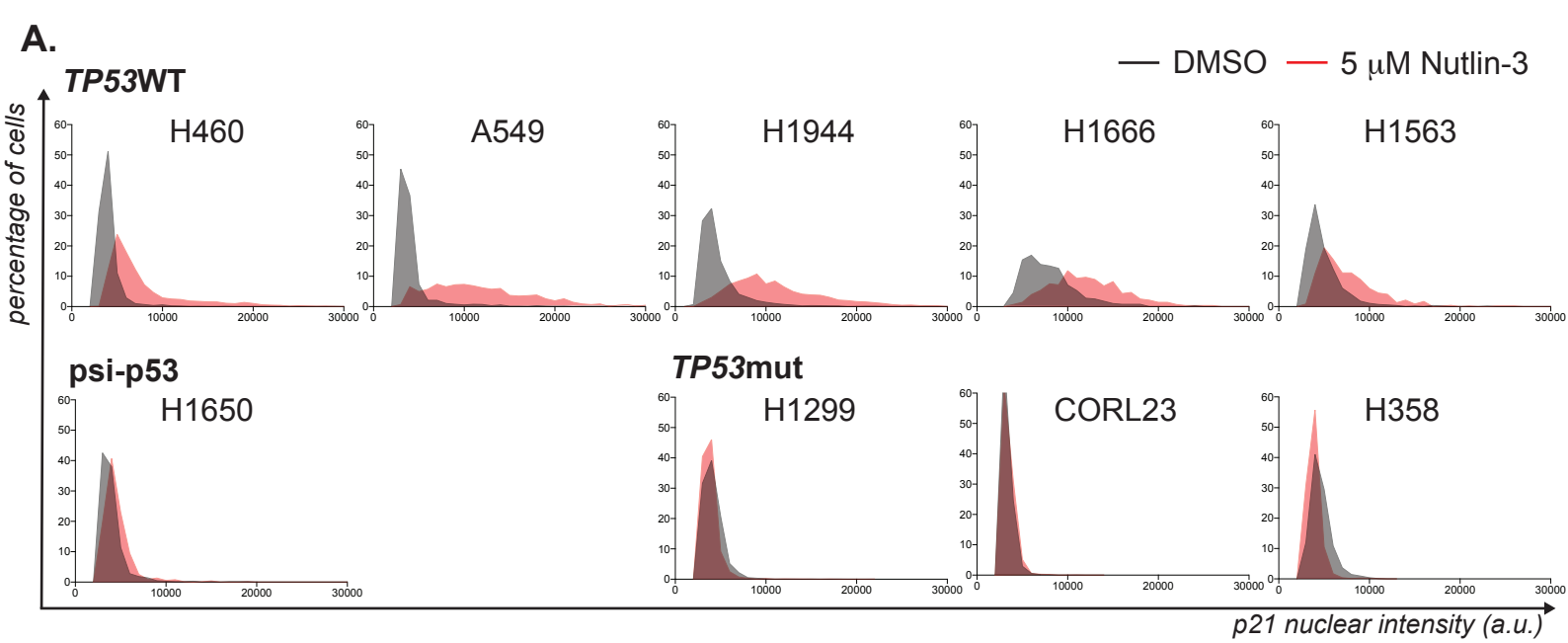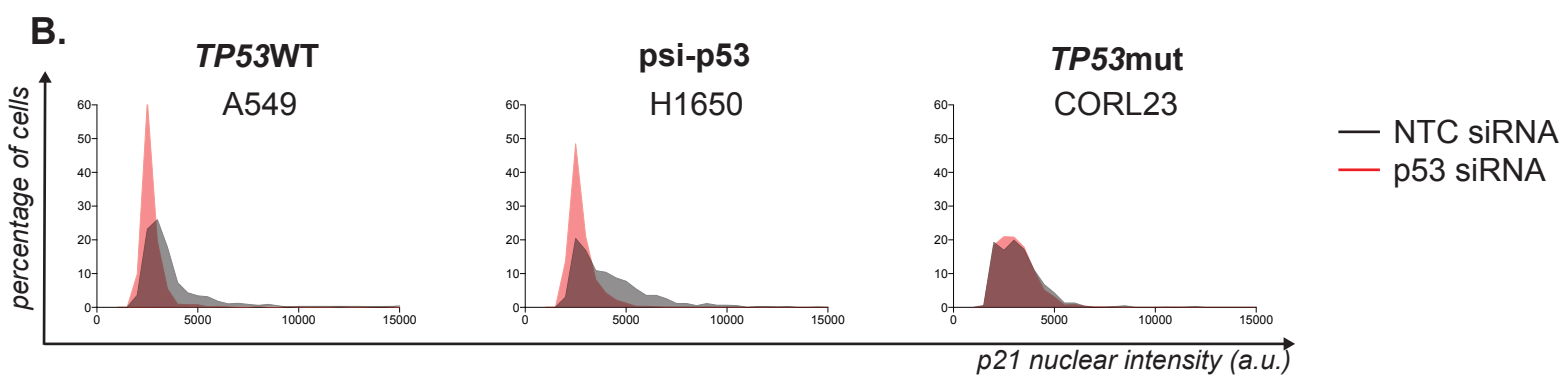

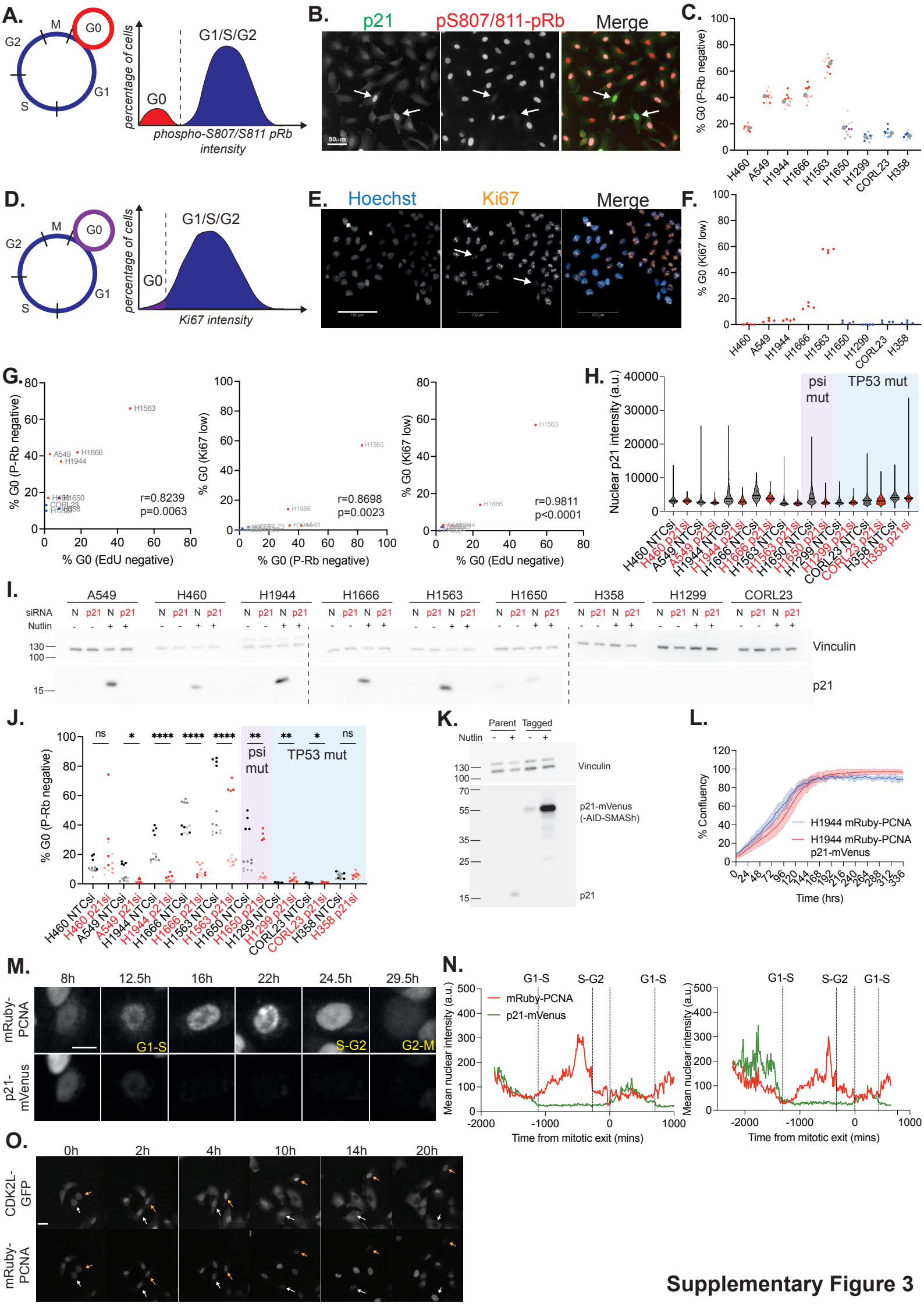

**A.**

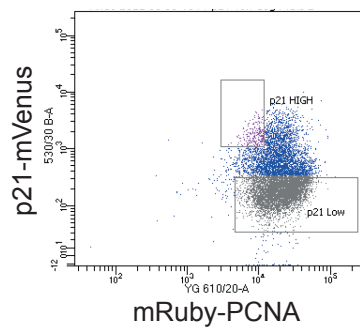

**B.**

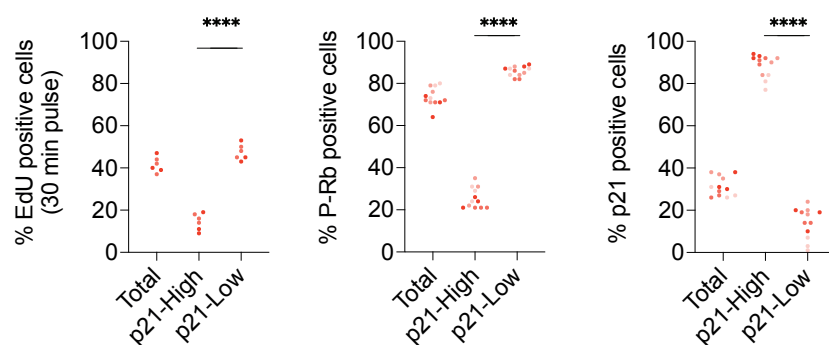

**C.**

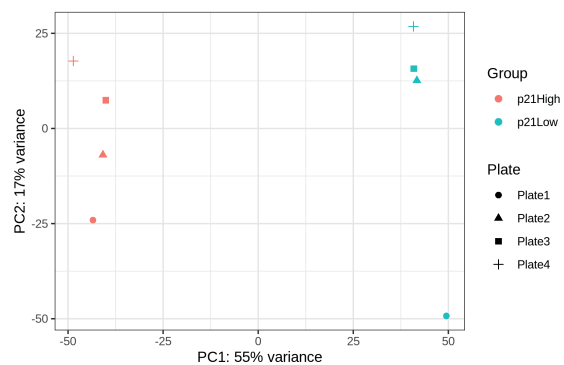

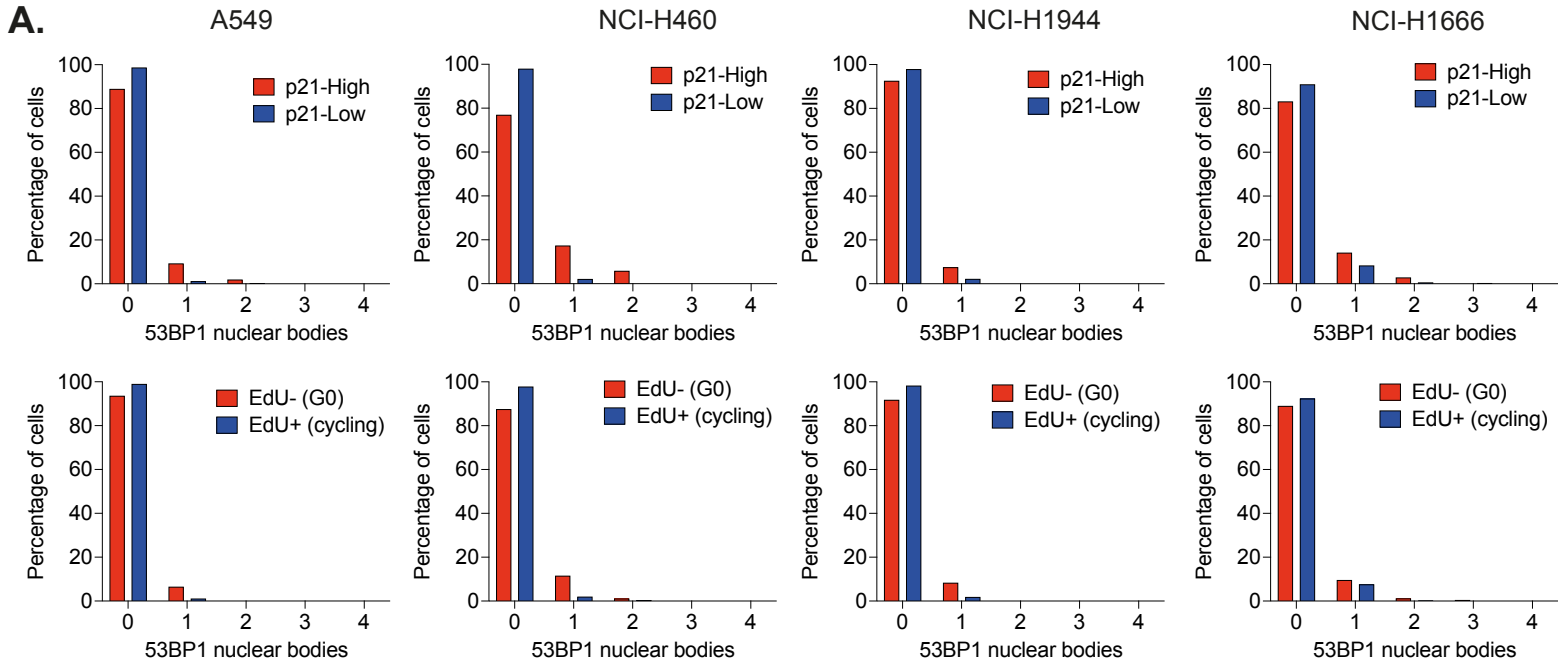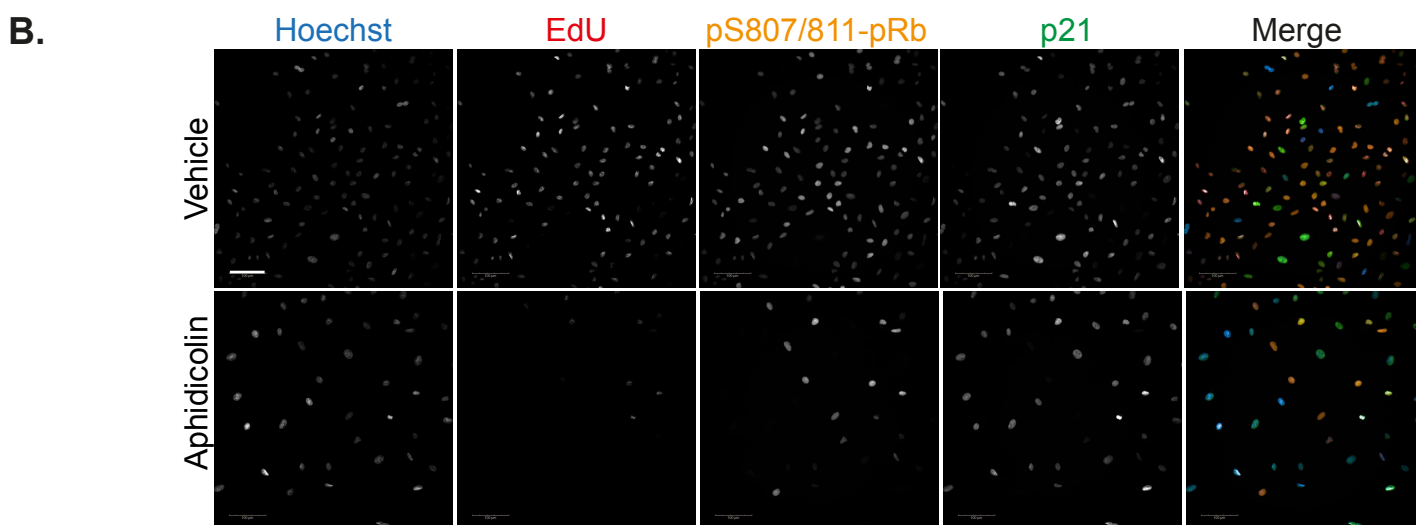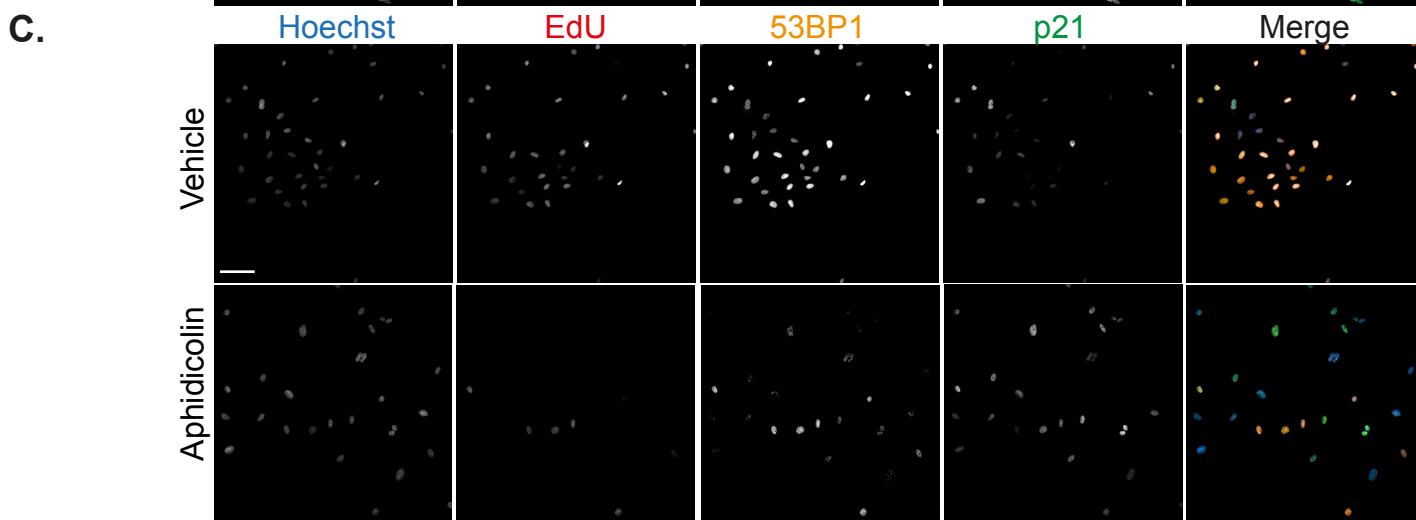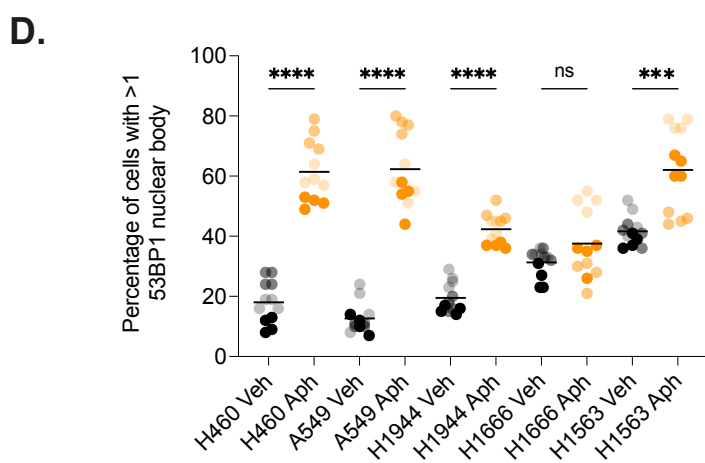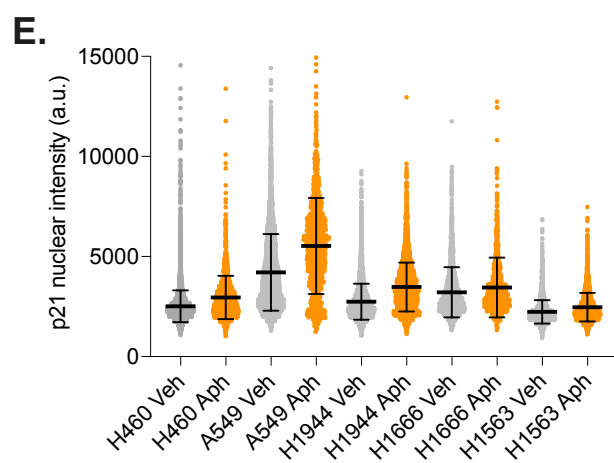

**Supplementary Figure 5**

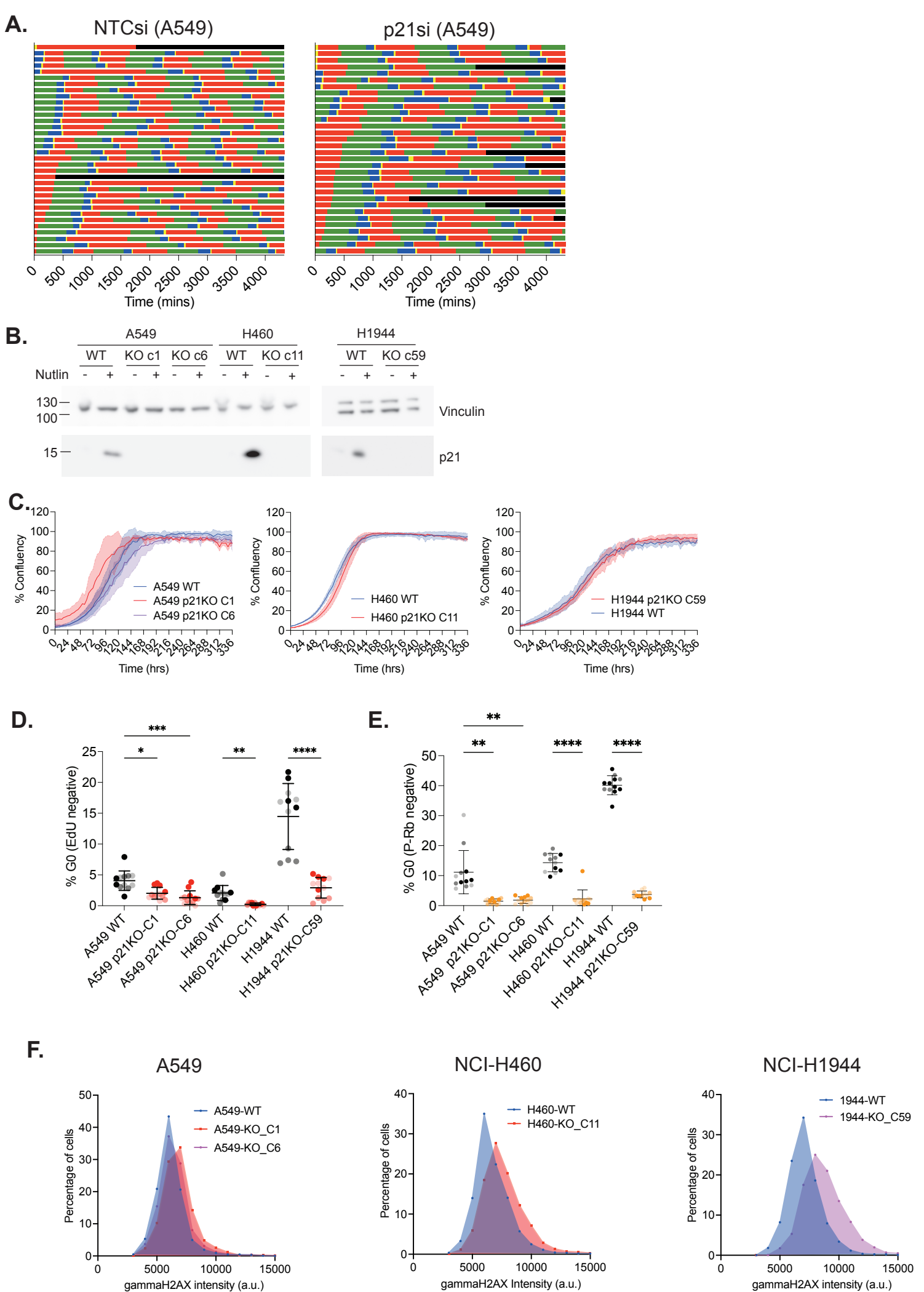

Supplementary Figure 6

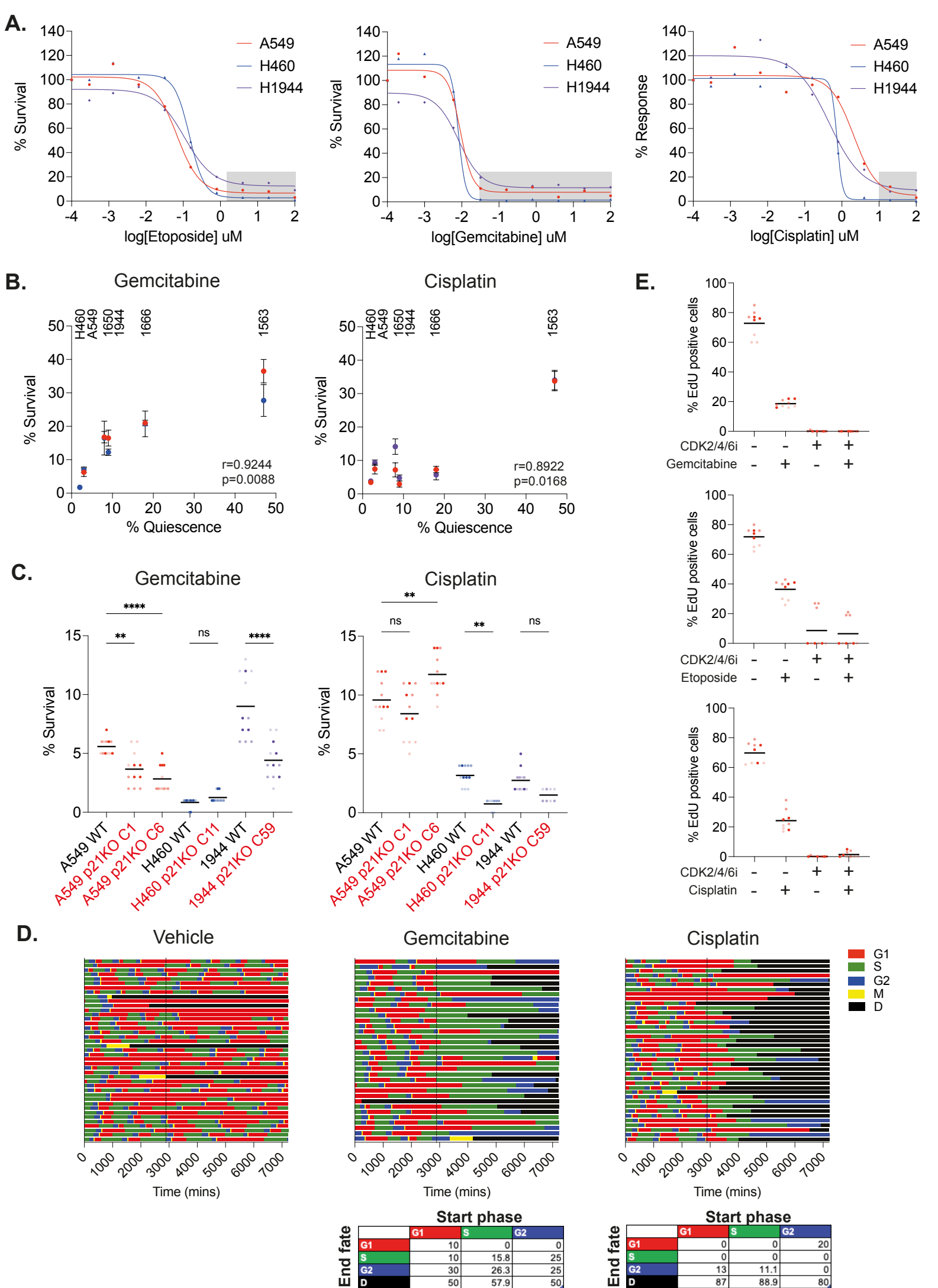

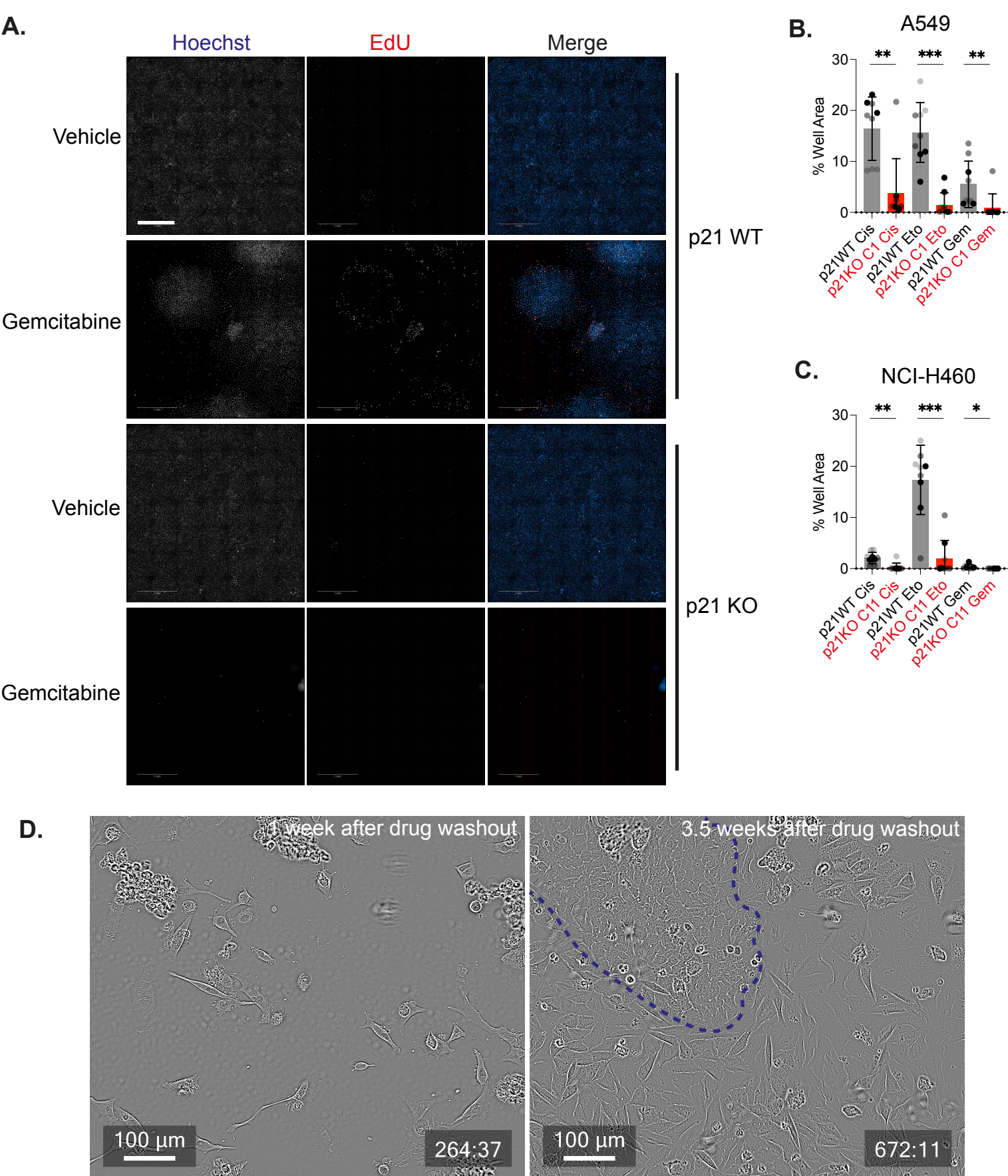

Supplement: Supplementary file 8 — Supplementary Figures [file 41416_2024_2928_MOESM8_ESM.pdf]
